# A 10-Year Follow-Up of Two-Incision and Modified Watson-Jones Total Hip Arthroplasty in Patients with Osteonecrosis of the Femoral Head

**DOI:** 10.1155/2017/8915104

**Published:** 2017-03-12

**Authors:** Shih-Jie Lin, Tsan-Wen Huang, Po-Chun Lin, Feng-Chih Kuo, Kuo-Ti Peng, Kuo-Chin Huang, Mel S. Lee

**Affiliations:** ^1^Department of Orthopaedic Surgery, Chang Gung Memorial Hospital, Chiayi, Taiwan; ^2^Change Gung University, Taoyuan, Taiwan; ^3^Department of Orthopaedic Surgery, Kaohsiung Chang Gung Memorial Hospital, Kaohsiung, Taiwan

## Abstract

Long-term data and information indicating whether minimally invasive surgery (MIS) approaches are safe and effective with total hip arthroplasty (THA) are lacking. Between 2004 and 2006, 75 patients with alcohol-related osteonecrosis of the femoral head (ONFH) who underwent 75 THAs with the two-incision approach were studied. The medical records, radiographic parameters, and functional outcomes were collected prospectively. All data were compared with those for matched patients who underwent a modified Watson-Jones (WJ) approach. THA using the two-incision approach was associated with longer operation time, more blood loss, more lateral femoral cutaneous nerve injury, and more periprosthetic femoral fractures (*p* < 0.05 for all four) than the modified WJ approach. The Harris Hip Score (HHS) and Western Ontario and McMaster University Osteoarthritis Index (WOMAC) increased significantly from the period preoperatively to 6 weeks postoperatively and thereafter up to the last follow-up in both groups. However, there were no significant differences in terms of radiographic parameters and functional outcomes between the two groups throughout the study period. Both the two-incision and the modified WJ approach provided satisfactory results and survival rates at a mean follow-up of 10.8 years. A prospective, randomized, large-scale cohort study is still warranted for evidence-based recommendations.

## 1. Introduction

Total hip arthroplasty (THA) is an effective surgery for patients with end-stage osteonecrosis of the femoral head (ONFH) [1]. However, the surgical approach is one of the main confounding factors for the outcome [2, 3]. Abnormal hip loading patterns have been noted after conventional THA and may affect longevity due to high cycle numbers [4]. Minimally invasive surgery (MIS) approaches to THA have been introduced in response to increased patient demands and expectations [2–8]. Early reports suggest that MIS lessens blood loss, pain, and hospital stay and results in early functional recovery compared with conventional approaches [5–7]. However, the merits of different MIS approaches are still controversial, and functional outcomes among the different surgical approaches vary [4, 8]. In general, MIS approaches can be divided into those using an abridged incision, such as transgluteal or posterolateral approaches, and those using a muscle-sparing approach, such as the two-incision and modified Watson-Jones (WJ) approaches [6–8]. From the technical perspective, the muscle-sparing approach provides adequate tissue tension and stability for THAs [9–11]. Better joint stability prevents microseparation or subluxation during gait cycles and avoids edge-loading-related accelerated polyethylene wear and sequential periprosthetic osteolysis and mechanical loosening [12, 13].

Concerns exist regarding the safety, efficacy, and longevity of MIS THA [4–8]. The available information from clinical reports is inadequate to suggest that surgeons should change from their standard approach [3]. Comparative studies of minimally invasive direct-anterior, minimally invasive direct-lateral, minimally invasive posterior, minimally invasive posterolateral, minimally invasive anterolateral, and two-incision approaches have been reported [14–18]. The inconsistency of the results may be partially due to the different surgical approaches (abridged incision or muscle-sparing approach) used with the study populations. While a few comparisons of the two-incision technique and the modified WJ technique have been reported, the literature on long-term outcome is sparse [4, 8]. The purpose of this study was to analyze the clinical outcomes and 10-year follow-up of ONFH patients who underwent THAs using the minimally invasive two-incision and modified WJ approaches.

## 2. Materials and Methods

This retrospective study was approved by the Ethics Committee and Institutional Review Board of our institution, and all patients provided signed informed consent.

The records of all patients who had undergone THA at our institution since 2004 were routinely entered into our database. After a clear explanation of the merits and disadvantages of different surgical approaches, the patients could choose the type of approach they wanted. We prospectively collected clinical data on age, gender, diagnosis, length of hospital stay, surgical approach used, total blood loss, and complications, as well as preoperative and postoperative radiographic and clinical functional assessments for each patient.

From 2004 to 2006, 262 patients with alcohol-related ONFH underwent a total of 316 metal-on-polyethylene THAs performed by a single surgeon. We manually identified patients who had unilateral hip involvement and chose to undergo a two-incision approach. The Fiber Metal Taper stem (Versys, Zimmer), used with a cementless press-fitting technique, and a highly cross-linked polyethylene-bearing surface were used in all THAs. Those patients with (a) previous surgeries on the hip joint, (b) a preexisting hip deformity, (c) bilateral THAs, and (d) incomplete medical records, radiographic analyses, or clinical functional assessments were excluded.

Seventy-five patients met our criteria. The study group consisted of 65 men and 10 women with a mean age of 44 years (range: 30 to 60 years) at the time of surgery. Age, gender, and date-of-surgery-matched patients with ONFH who had undergone THAs using the same prostheses and a modified WJ approach performed by the same surgeon were used as controls. To determine adequate sample size, an* a priori* power analysis using the hypothesis test with a power of 90% and a significance of 0.05 was performed. According to Achten et al. [19], 48 hips were required per group to detect a difference of 10 points in the Harris Hip Score (HHS) (estimated standard deviation of 15). A cut-off value was selected because a difference of 10 points was suggested as the minimal clinically important difference.

All patients enrolled in this study were treated with the same protocol. On the basis of the standard of care following cementless THA, the patients were encouraged to ambulate with partial weight-bearing as soon as possible after surgery, under the supervision of a physical therapist. All patients used crutches or a walker with full weight-bearing for 6 weeks and a cane when needed thereafter.

Radiological assessments included the cup inclination, the cup anteversion, the presence of radiolucent lines in the prosthesis-bone interface, the alignment and canal fill ratio of the femoral stem, and the limb-length discrepancy, as previously described [20–22]. The radiographic assessments were reviewed and analyzed by an independent surgeon who was blinded to the groupings and patient demographic data. Intraobserver reliability was assessed according to the method described by Konigsberg et al. [23] and was rated as good to very good. Clinical results were assessed using the HHS [24] and Western Ontario and McMaster University Osteoarthritis Index (WOMAC) [25], beginning preoperatively and at intervals of 6 weeks, 3 months, and 6 months and yearly after the surgery. All functional outcomes were assessed by an independent observer who was also blinded to the groupings and patient demographic data.

Complications were recorded. Any medical or surgical event that compromised the clinical recovery of the patients, such as wound infection, venous thromboembolism (VTE), neurovascular injury, fracture, dislocation, implant malposition, implant size mismatch, or early loosening, was defined as an adverse event.

### 2.1. Statistical Analysis

Statistical analysis was performed by an independent statistician using the SPSS for Windows statistical package (version 17.0, SPSS, Chicago, Illinois). Differences among patients who underwent the two-incision approach and control patients were examined using the *χ*^2^ test for categorical variables, a nonparametric test for ordinal variables, and the *t*-test for continuous variables. Significance was set at *p* < 0.05.

## 3. Results

The demographic and perioperative variables of patients undergoing the two-incision approach and control patients are shown in Table 1. Patients undergoing the two-incision approach had a longer operation time (160 minutes versus 117 minutes, *p* < 0.001) and more blood loss (719 mL versus 366 mL, *p* < 0.001) than did patients in the WJ group. With regard to length of hospital stay and wound length, there was no significant differences between the 2 groups.

Radiological analysis revealed no significant differences in cup inclination, cup anteversion angles, stem alignment, canal fill ratio, or limb-length discrepancy. The percentages of procedures that had ideal positioning were similar between the 2 groups (Table 2).

Seven patients that had periprosthetic fracture (6 in the two-incision group and 1 in the WJ group) were excluded from the final analysis because there would be different rehabilitation protocols and influences on outcome assessment. The remaining hips were included in the final functional analysis. There were no significant differences in HHS and WOMAC scores in either group before surgery. The HHS and WOMAC scores increased significantly from baseline to 6 weeks and thereafter up to the most recent follow-up in both groups (*p* < 0.001). The 2 groups did not differ in functional outcomes during the entire study period (Table 3).

There was no difference in complications between the 2 groups, except for lateral femoral cutaneous nerve palsy (*p* < 0.001) and periprosthetic fracture (*p* = 0.049). A summary of complications during the 10-year follow-up period revealed that no deep infection, VTE, or implant size mismatch had occurred in either group. Both groups showed similar findings with regard to cup loosening, stem loosening, superficial wound infection, and dislocation of the hip. The overall complication rate was significantly higher in the two-incision group (41.3%) (*p* < 0.001) (Table 4).

Twenty hips in the two-incision group were complicated with lateral femoral cutaneous nerve palsy in the thigh. At the last follow-up, 3 of them still had residual symptoms. However, there were no nerve palsy complications in the WJ group, a significant difference (*p* < 0.001). One hip in the WJ group sustained an intraoperative periprosthetic fracture of the proximal femur that needed additional cerclage wire fixation. In contrast, 6 hips in the two-incision group sustained an intraoperative periprosthetic femoral fracture, 4 of which were fixed with cerclage wires during the index operation and 2 of which were detected in the postoperative stage when the femoral stem subsided. The latter 2 fractures needed a second surgical procedure to revise the femoral component.

**Figure 1:**
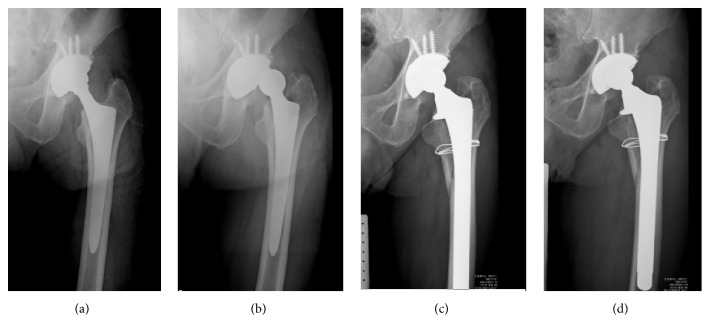
A 62-year-old male with alcohol-related ONFH underwent THA with a two-incision approach. (a) Immediate postoperative left hip anteroposterior view. (b) Loosening of femoral stem and hip dislocation occurred in the postoperative 6 months. (c) Revision of femoral component with long stem was performed. (d) Ten years later, the hip remains with good stability and adequate position.

One loose cup was found in the WJ group in the late stage. Two femoral stems in the two-incision group but none in the WJ group were loose. The loose femoral stems, both of which were revised, were diagnosed 6 and 9 months after the index operation (Figure 1). Using component revision as the endpoint for survival analysis, the survival rate for cups was 100% in the two-incision group and 98.7% in the WJ group. The survival rate for stems was 97.3% in the two-incision group and 100% in the WJ group. Three hips in the two-incision group had superficial wound infection, and all were successfully treated with antibiotics immediately; there was no wound infection in the WJ group. There was one hip dislocation, a posttrauma posterior dislocation, in the WJ group 3 months after the index operation. Closed reduction was successful, and there were no additional dislocations (Table 4).

## 4. Discussion

The most important finding in this investigation is that use of the modified WJ approach for THA in ONFH hips resulted in shorter operation time, less blood loss, and a lower incidence of lateral femoral cutaneous nerve injury and periprosthetic femoral fracture compared to the two-incision approach. However, there was no statistically significant difference between the 2 groups in radiographic parameters, functional outcomes, and survival rate at a mean follow-up of 10.8 years.

MIS THA has gained popularity in recent years. To date, however, no consensus exists regarding the merits, safety, and efficacy of MIS THA [4–8]. Nevertheless, questions have been raised regarding whether iatrogenic complications such as fractures or nerve injuries can be prevented and whether components can be placed in their correct position through small incisions [11]. Comparative studies of abridged incision approaches and muscle-sparing approaches have been reported, but little has been published regarding comparisons of muscle-sparing techniques [4–8]. In addition, long-term data regarding the longevity of MIS THA is lacking, as is information indicating whether MIS approaches are safe and effective with THA [5–8].

There are 2 comparative studies on two-incision THA and modified WJ THA in the literature. In 2012, we published a prospective randomized study on patients who had a two-incision THA in one hip and a modified WJ THA in the other to investigate the efficacy of the 2 techniques and found no significant difference in the radiographs and clinical outcomes of the patients [8]. However, several limitations must be acknowledged. First, the study was limited by its short-term clinical follow-up. Second, only 20 patients (40 hips) were studied. Third, the reasons for undergoing THA included both ONFH and osteoarthritis (OA). In the second study, Foucher et al. [4] conducted a randomized controlled trial involving 32 patients with OA. The purpose of the trial was to determine the efficacy of the two-incision and modified WJ approaches, individually, during the first postoperative year. No significant differences were detected between the 2 techniques in terms of gait analysis and time-course of recovery. However, similar to our study, the evidence was limited by the small number of patients and the short-term follow-up.

In this study, the operation time was longer in the two-incision group. The surgical field could not be fully visualized during implantation of the femoral stem and repeated evaluation of its position by intraoperative fluoroscopy may have resulted in longer operation times and more blood loss in this group. Lateral femoral cutaneous nerve injury occurred only in the two-incision group, with an incidence rate of 26.7%, which is comparable to the 24.7% rate in another study [26]. This recognized risk is associated with the various anterior approaches to the hip joint, including the two-incision approach [26, 27]. To avoid this complication, some surgeons use the intermuscular interval between the gluteus medius and the tensor fascia latae (the WJ interval) in their two-incision techniques [28]. Our findings are compatible with their results: the WJ group had fewer cases with lateral femoral cutaneous nerve injury.

Periprosthetic fractures in the perioperative or early postoperative stage are primarily iatrogenic [29–31]. In this study, we had a periprosthetic femoral fracture rate of 8.0% in the two-incision group (1.3% in the WJ group), which was higher than the 2.5% fracture rate reported in a Chinese study that used a cementless press-fit technique with ONFH hips [32]. In ONFH hips, the lesion can be extensive and involve the area below the lesser trochanter in 5% to 11% of hips [32–34]. In addition, patients with ONFH have been reported to be “at risk” for abnormal hip anatomy with a low neck-shaft angle and high femoral neck version. These anatomical variations and the limited surgical exposure might cause reconstruction difficulties and possibly contribute to more surgeon-related complications when using minimally invasive approaches [35]. For cup implantation, the two-incision and modified WJ approaches are similar, and the only difference is in the different intermuscular interval used. For implantation of the femoral stem, however, the 2 techniques are quite different. One incision for the cup and another for the stem should theoretically be feasible for implanting a prosthesis and precluding complications like a periprosthetic fracture. However, the femoral fracture rate was higher in our two-incision group. We hypothesized that this was because the surgical field could not be fully visualized during stem implantation with the two-incision approach; this technique has an inherently high risk of complications that is difficult to minimize, even by surgeons with advanced proficiency in the procedure [36, 37].

There was a 5.3% revision rate for the stem in the two-incision group, including 2 periprosthetic fractures and 2 with aseptic loosening of the stem. With stem revision as the endpoint, survivorship in both groups for any reason was 97.3%, and for aseptic loosening, it was 98.6% at 10 years. This is compatible with reports [38–41] on the durability of conventionally performed cementless THAs in cohorts with ONFH.

This study has some limitations. First, this was a retrospective study, and the cases were not randomized. However, all of the surgeries were done by the same surgeon using the same protocol and implants, which decreases the effects of some confounding factors. Second, the case number was modest: 75 hips in each group. One study [19], using the HHS scoring system to calculate a sample size with a power of 90% and a significance of 0.05 to detect a difference of 10 points in the HHS score (estimated SD of 15), found that 48 hips were required per group. Although our sample size was more than adequate to detect a difference in HHS, this study may still be underpowered to demonstrate significant differences. Finally, only clinical and radiological assessments were done. Functional analyses (gait and muscle strength) and other kinematic studies that might have explored more of the risks and benefits of different surgical approaches [8, 42] were not done.

In conclusion, both the two-incision and modified WJ approach provided satisfactory clinical results and survival rates at a mean follow-up of 10.8 years. However, there were high incidences of lateral femoral cutaneous nerve injury and periprosthetic femoral fracture when using the two-incision technique. We now routinely use the modified WJ approach for THAs in patients with ONFH. A prospective, randomized, large-scale cohort study is still warranted to provide evidence-based recommendations for patients with ONFH.

## Figures and Tables

**Table 1:** Demographic data on the two-incision and Watson-Jones groups.

Demographic information	Two-incision group	WJ group	*p* value
Age (years)	44 ± 8	44 ± 8	Matched
Gender (male : female)	65 : 10	65 : 10	Matched
Side of operation (number of hips)			0.988
Right	42 (56.0%)	41 (54.7%)	—
Left	33 (44.0%)	34 (45.3%)	—
Operation time (min)	160 ± 41	117 ± 36	<0.001^*∗*^
Perioperative blood loss (mL)	719 ± 423	366 ± 208	<0.001^*∗*^
Length of hospital stay (day)	5 ± 1	5 ± 2	0.399
Wound length (cm)	10 ± 1	9 ± 2	0.677

WJ = Watson-Jones.

Values are shown as mean (standard deviation) or as *n* (%).

*p* values for between-group comparisons were determined by the chi-squared test and Fisher's exact test for categorical variables and Student's *t*-test for continuous variables.

^*∗*^Statistically significant (*p* < 0.05).

**Table 2:** Comparison of radiographic results in the two-incision and Watson-Jones groups.

	Two-incision group	WJ group	*p* value
Cup inclination angle (deg)	44 ± 5	45 ± 4	0.052
Number of outliers	4 (5.3%)	7 (9.3%)	0.325
(cups with angle of ≤30° or ≥50°)			
Cup anteversion (deg)	17.5 ± 7.7	15.7 ± 6.4	0.105
Number of outliers	17 (22.7%)	9 (12.0%)	0.099
(cups with angle of ≤5° or ≥25°)			
Stem alignment (deg)	Valgus	Valgus	0.127
	0.1 ± 1.1	−0.2 ± 1.2	
Canal fill ratio (%)	94.0 ± 4.1	94.0 ± 4.2	0.661
Limb-length discrepancy (mm)	0.7 ± 1.5	0.8 ± 2	0.510

Values are shown as mean (standard deviation) or as *n* (%).

*p* values for between-group comparisons were determined by the chi-squared test and Fisher's exact test for categorical variables and Student's *t*-test for continuous variables.

**Table 3:** Functional results of the two-incision and Watson-Jones groups.

	Two-incision group	WJ group	*p* value
Harris Hip Score			

Preoperative	57 ± 12	56 ± 11	0.151
6 weeks	90 ± 6	91 ± 6	0.982
3 months	94 ± 5	94 ± 5	0.961
6 months	96 ± 4	96 ± 4	0.933
1 year	98 ± 3	97 ± 4	0.868
Last follow-up	94 ± 6	93 ± 5	0.799

Western Ontario and McMaster University Osteoarthritis Index			

Preoperative	58 ± 9	56 ± 8	0.222
6 weeks	91 ± 5	89 ± 8	0.956
3 months	95 ± 5	96 ± 5	0.971
6 months	96 ± 4	99 ± 4	0.854
1 year	99 ± 5	99 ± 3	0.822
Last follow-up	95 ± 4	96 ± 5	0.868

WJ = Watson-Jones.

Values are shown as mean (standard deviation).

*p* values for between-group comparisons were determined using Student's *t*-test.

**Table 4:** Complications in the two-incision and Watson-Jones groups.

	Two-incision group	WJ group	*p* value
Lateral femoral cutaneous nerve palsy	20 (26.7%)	0	<0.001^*∗*^
Periprosthetic femoral fracture	6 (8.0%)	1 (1.3%)	0.049^*∗*^
Cup loosening	0	1 (1.3%)	0.493
Stem loosening	2 (2.7%)	0	0.242
Superficial wound infection	3 (4.0%)	0	0.118
Dislocation of the hip	0	1 (1.3%)	0.493
Hips with complications (*n*)	31 (41.3%)	3 (4.0%)	<0.001^*∗*^

Values are shown as mean (standard deviation) or as *n* (%).

*p* values for between-group comparisons were determined by the chi-squared test and Fisher's exact test.

^*∗*^Statistically significant (*p* < 0.05).
